# Genistein Improves the Cytotoxic, Apoptotic, and Oxidative-Stress-Inducing Properties of Doxorubicin in SK-MEL-28 Cancer Cells

**DOI:** 10.3390/medicina61050798

**Published:** 2025-04-25

**Authors:** Andrea Roman, Andrei Motoc, Iasmina Marcovici, Cristina Dehelean, Laura Nicolescu, Casiana Boru

**Affiliations:** 1 Faculty of Medicine, “Vasile Goldis” Western University of Arad, 94 Revolutiei Blvd., 310130 Arad, Romania; 2 Faculty of Medicine, “Victor Babes” University of Medicine and Pharmacy, Eftimie Murgu Square No. 2, 300041 Timisoara, Romania; 3 Faculty of Pharmacy, “Victor Babes” University of Medicine and Pharmacy, Eftimie Murgu Square No. 2, 300041 Timisoara, Romania; 4 Research Center for Pharmaco-Toxicological Evaluations, Faculty of Pharmacy, “Victor Babes” University of Medicine and Pharmacy, Eftimie Murgu Square No. 2, 300041 Timisoara, Romania

**Keywords:** cutaneous melanoma cells, genistein, doxorubicin, cytotoxicity, apoptosis, oxidative stress, keratinocytes, non-irritant effect

## Abstract

*Background and Objectives:* Cutaneous melanoma (CM) poses a continuous challenge in oncology due to the developing resistance to available treatments. Doxorubicin (DOX) is noted as one of the most effective chemotherapeutics, although associated toxicity and resistance limit its use in CM treatment. Consequently, DOX has become a promising candidate for combination therapies targeting this neoplasm. Genistein (GEN) gathered significant attention due to its anti-neoplastic properties and ability to enhance the effects of DOX against several cancers, yet this association remains underexplored in CM. Therefore, this study investigated the combination therapy regimen comprising GEN and DOX in terms of anti-melanoma activity and safety profile. *Materials and Methods*: The in vitro experiments were performed on SK-MEL-28 and HaCaT cells. Cell viability was determined using MTT assay. Cell morphology and confluence were inspected microscopically. Nuclear and cytoskeletal aspects were assessed via immunofluorescence. Apoptosis and oxidative stress were quantified through caspase activity and intracellular reactive oxygen species (ROS) production, respectively. The irritant effect was evaluated on the chorioallantoic membrane. *Results*: The results revealed that the combination of GEN 10 µM with DOX (0.5 and 1 µM) provided augmented cytotoxic events (e.g., reduced cell viability, altered cell morphology and confluence, apoptotic-like impairments in nuclear shape and cytoskeletal network, increased caspases-3/7 and -9 activity, and elevated ROS) in SK-MEL-28 cells, compared to individual treatments, and exerted a strong synergistic interaction. Simultaneously, GEN 10 µM efficiently surpassed the toxic effects (e.g., viability and confluence loss, hypertrophy, and cytoskeletal condensation) of DOX (0.5 and 1 µM) in HaCaT cells. In ovo, GEN 10 µM + DOX 1 µM treatment was classified as non-irritant. *Conclusions*: These findings stand as one of the first contributions revealing the beneficial therapeutic interplay between GEN and DOX at physiologically achievable concentrations that resulted in elevated anti-tumor properties in CM cells and alleviated toxicity in keratinocytes.

## 1. Introduction

Cutaneous melanoma (CM) represents the deadliest form of skin cancer, and it is the most common melanoma subtype, accounting for over 90% of all diagnosed cases [1,2]. The complexity and aggressiveness of the disease result mainly from its high level of heterogeneity and increased metastatic potential [3]. The CM development was associated with both environmental and genetic risk factors, with the main trigger of carcinogenesis being the exposure to ultraviolet radiation (UV) from natural or artificial sources [2]. Despite the significant advances made in the comprehension of CM genetics, biology, and treatment, the curative options available for the management of this malignancy remain limited, especially in the advanced stages of the disease [3]. Currently, chemotherapy remains one option for the treatment of metastatic CM, although no particular drug regimen has been found to provide an overall survival benefit to treated patients, with the major impediments of the current chemotherapeutics remaining the acquirement of multidrug resistance and various toxic effects [4,5,6]. Additionally, the last decade has witnessed the unprecedented revolutionizing of CM treatment through the development of immune checkpoint inhibitors (specifically cytotoxic T-lymphocyte associated protein-4 (CTLA-4) and programmed cell death protein-1 (PD-1) inhibitors) and targeted therapies (v-Raf murine sarcoma viral oncogene homolog B1 (BRAF) and mitogen-activated protein kinase (MEK) inhibitors) [7]. However, it has been well documented that the duration of the response to targeted therapy is limited, and the clinical benefits after immunotherapy remain modest [4], as intrinsic or acquired resistance were also reported for these advanced tumor-targeted treatments [6].

A substantial agreement has been raised on the fact that the development of novel strategies able to overcome the intrinsic drug resistance of CM cells would significantly improve the survival rate of patients undergoing therapy [8]. Recently, the concept of combination therapy, a treatment modality that associates two or more therapeutic agents in one drug, has been extensively evolving as the cornerstone of cancer treatment, distinguishing itself from monotherapy due to its higher efficacy, patient compliance, and treatment adherence, as well as lower drug doses and adverse effects [9,10]. Several combinatorial approaches for CM management have been reported and comprehensively reviewed elsewhere [11]. In this context, doxorubicin (DOX) has become a focal point of interest for CM combinatorial treatments, as it constitutes one of the main components of the anti-tumor therapy regimens and one of the most effective drugs ever developed, being currently available on the market [5,6,12]. DOX exerts its pleiotropic antineoplastic effects through several mechanisms, including cell death (e.g., apoptosis, autophagy, pyroptosis, etc.) induction, reactive oxygen species (ROS) generation, and DNA damage [12]. With regard to CM, the therapeutic benefits of DOX are limited at present due to the developed drug resistance [5], unsustainable efficiency, and undesirable adverse reactions [13]. In response to these issues, several studies have been conducted to elevate treatment efficacy and abolish resistance. For instance, Licarete et al. recently described a novel approach in overcoming the resistance of DOX in CM through liposomal combination therapy. Their study indicated that the association of a liposomal formulation of prednisolone disodium phosphate with a liposomal formulation of DOX results in enhanced cytotoxicity, anti-angiogenic, and anti-metastatic effects in B16F10 CM in vitro and in vivo models [14]. Another study demonstrated the improved anti-cancer efficiency obtained following the association between DOX and caffeine in B16F10 CM tumors, evidenced by the induction of immuno-genic cell death and T-cell infiltration [15]. Dorasamy et al. investigated the ability of brequinar sodium to potentiate the anti-neoplastic activity of DOX against CM. They found that this combination therapy produced synergistic and additive growth inhibition in A375 CM cells, while also causing 90% CM tumor regression in vivo [16]. Additionally, a recent meta-analysis presented highly relevant studies regarding the synergistic action of the cold atmospheric plasma and doxorubicin in melanoma [17].

In particular, plant-derived compounds, highly treasured as a valuable source for drug discovery and development, gained outstanding attention for combination-therapy-based cancer treatment, enhancing the efficacy of conventional chemotherapeutics [18,19]. By chemosensitizing tumor cells to traditional drugs, such bioactive agents delay the appearance of drug resistance, enhance the antitumor efficacy, limit their side effects, and improve the outcome of cancer patients [20,21]. This innovative strategy was successfully applied for enhancing the anti-tumor effects of DOX against CM. A suitable example in this regard is the study conducted by Mittal et al., who discovered that the isoquinoline alkaloid berberine, naturally found in plant species such as *Tinospora cordifolia*, efficiently suppressed cell growth, triggered cell death, and caused cell cycle arrest in B16F10 CM cells, while also reducing B16F10 tumor volume and weight in vivo when used as combinatorial treatment with DOX [22]. In alignment with these preceding findings, Ebbert and collaborators demonstrated the beneficial outcomes of the addition of the natural chalcone cardamonin, distributed within *Alpinia* species, to DOX treatment such as a selective cytotoxic effect against A375 CM cells, and alleviated DOX-induced toxic effects in human dermal fibroblasts and rat cardiac myoblasts (H9c2) [6]. A nature-derived compound that proved highly beneficial when co-administered with DOX is genistein (GEN), a phytoestrogen comprising an isoflavone structure and widely distributed in soy-based products. GEN stands out due to its multiple biological effects, including estrogenic properties and antitumor activity by modulating apoptotic cell death, cell cycle, metastasis, and angiogenesis [23,24,25]. The anti-melanoma properties retained by GEN were also extensively emphasized [25,26]. Moreover, to date, several reports have outlined not only the efficacy of GEN in counteracting the side effects associated with DOX chemotherapy (i.e., cardiotoxicity [27]), but also its ability to enhance the anticancer properties of DOX against several neoplasms (e.g., breast, prostate [28,29]), becoming a promising candidate for inclusion in DOX-based combination therapy regimens for tumor ablation.

Nonetheless, despite this previous comprehensive research highlighting the value of the association of GEN and DOX in chemotherapy, its potential application in CM remains mostly unexplored. Therefore, the present study was undertaken to assess the potential improved anti-tumor efficiency of the genistein–doxorubicin (GEN–DOX) combinatorial treatment in SK-MEL-28 CM cells. Moreover, taking into account the many side effects exerted by DOX, the assessment of potential cytoprotective activity of GEN against its damage in HaCaT keratinocytes, as well as of the non-irritant effect of the GEN–DOX treatment on the vascularized chorioallantoic membrane (CAM), were additional interests for this research.

## 2. Materials and Methods

### 2.1. Materials

The following materials were bought from Sigma Aldrich (St. Louis, MO, USA): genistein, doxorubicin hydrochloride, MTT Kit, 4′,6-diamidino-2-phenylindole (DAPI), and Trypan blue solution 0.4%. The cell culture media (DMEM and EMEM), trypsin-EDTA solution, and fetal bovine serum (FBS) were purchased from ATCC (American Type Cell Collection, Lomianki, Poland). The Caspase-Glo^®^ 3/7, Caspase-Glo^®^ 9, and ROS-Glo™ H_2_O_2_ assay kits were achieved from Promega Corporation (Madison, WI, USA). Ultrapure distilled water and specific antibodies (TexasRed-X Phalloidin, alpha tubulin monoclonal antibody (B-5-1-2), and goat anti-mouse IgG (H+L) secondary antibody–Alexa Fluor™ 488) were obtained from Thermo Fisher Scientific Inc. (Waltham, MA, USA). Bovine serum albumin (BSA) was delivered by Cell Signaling Technology (Danvers, MA, USA). The antibiotic solution (penicillin 100 U/mL–streptomycin 100 µg/mL) and dimethyl sulfoxide (DMSO) were provided by PanBiotech (Aidenbach, Germany).

### 2.2. Instruments

Cytation 5 (microplate reader), Lionheart FX (automated microscope), and Gen5™ Microplate Data Collection and Analysis Software (Version 3.14) were acquired from BioTek Instruments Inc. (Winooski, VT, USA). The Olympus IX73 microscope and the cellSens Dimensions v.1.8. Software were obtained from Olympus (Tokyo, Japan).

### 2.3. Cell Culture Method

The present study was performed on two cell lines received from ATCC and CLS, respectively, as frozen vials: SK-MEL-28 (HTB-72™): human CM cells grown in EMEM medium, and HaCaT (300493): immortalized human keratinocytes cultured in DMEM medium. Both media were supplemented with FBS (10%) and a penicillin–streptomycin mixture (1%). During all experiments, the cells were maintained in a humidified incubator for cell culture, at 37 °C and 5% CO_2_.

### 2.4. Cell Viability Assessment–The MTT Test

The impact of GEN (5, 10, and 25 µM), DOX (0.5, 1, and 2.5 µM), and GEN 10 µM + DOX (0.5, 1, and 2.5 µM) on cell viability was assessed after 24 h of stimulation by conducting the MTT assay, as previously presented in a recent study [30]. To obtain the stock solutions for testing, GEN was dissolved in DMSO, while DOX was dissolved in ultrapure distilled water. Before treatment, the stock solutions were diluted in culture media (DMSO concentration did not exceed 0.5%). First, SK-MEL-28 and HaCaT cells were cultured in 96-well plates, at a density of 10^4^ cells per well, and left to adhere until a proper confluence was reached (70%). At the end of the treatment period, the culture media were changed, and MTT reagent (10 µL/well) was added. The plates were incubated at 37 °C and 5% CO_2_ for 3 h before the addition of 100 µL/well of solubilizing solution and incubation of the plates for half an hour at room temperature. The absorbance was read at 570 nm using Cytation 5. Cell viability was normalized to control and determined by applying the following formula:

$$\mathrm{Cell}\;\mathrm{Viability}\;(\%)=\frac{Absorbance\;of\;treated\;cells}{Absorbance\;of\;Control}\;\times \;100$$

The experiment was conducted in triplicate. Control indicates cells without GEN, DOX, or GEN–DOX treatment.

### 2.5. Combination Index Calculation

The interaction type (i.e., synergistic, additive, or antagonistic) between GEN and DOX and the combination index (CI) values were determined by applying the Chou–Talay method and using the CompuSyn software version 1.0 (ComboSyn, Inc., Paramus, NJ, USA). The interpretation of the results was performed according to previous publications [31,32].

### 2.6. Bright-Field Evaluation of Cell Morphology

For this assay, the cells were cultured in clear 96-well plates at a density of 10^4^ cells/well. The influence of GEN 10 µM, DOX (0.5 and 1 µM), and GEN 10 µM + DOX (0.5 and 1 µM) treatment on the SK-MEL-28 and HaCaT cells’ morphology after a 24 h exposure was evaluated by imaging the treated cells in bright-field, using the Olympus IX73 inverted microscope and the cellSens Dimensions v.1.8. software at a magnification of 20×. The experiment was conducted in triplicate. Control indicates cells without GEN, DOX, or GEN–DOX treatment.

### 2.7. Label-Free Quantification of Cell Confluence

To quantify the effect of GEN 10 µM, DOX (0.5 and 1 µM), and GEN 10 µM + DOX (0.5 and 1 µM) treatment on cellular confluence, SK-MEL-28 and HaCaT cells were photographed after the 24 h exposure at a magnification of 4×, on the Lionheart FX microscope. To measure the cell confluence values, the taken images were processed and analyzed with the Cell Analysis Tool included in the Gen5™ Microplate Data Collection and Analysis Software (Version 3.14), according to the manufacturer’s indications. The experiment was conducted in triplicate. Control indicates cells without GEN, DOX, or GEN–DOX treatment.

### 2.8. Immunofluorescence Imaging of Nuclei and Cytoskeletal Filaments

The potential impact of GEN 10 µM, DOX (0.5 and 1 µM), and GEN 10 µM + DOX (0.5 and 1 µM) treatment on the aspect of nuclei, F-actin, and tubulin in SK-MEL-28 and HaCaT cells was investigated by immunofluorescence microscopy. For this study, the cells were grown in black 96-well plates (10^4^ cells/well), treated with GEN 10 µM, DOX (0.5 and 1 µM), and GEN 10 µM + DOX (0.5 and 1 µM) for 24 h, fixed with a paraformaldehyde solution (4% in PBS) for 15 min at room temperature, permeabilized with Triton-X solution (0.1% in PBS) for 15 min at room temperature, and treated with bovine serum albumin (BSA) solution (1%) for 45 min at room temperature. F-actin was visualized using the TexasRed-X Phalloidin (dilution 0.5:200 in 0.1% BSA, 30 min incubation at room temperature). Tubulin was stained using alpha tubulin monoclonal antibody (B-5-1-2) (dilution 1:1000 in 0.1% BSA, incubation for 4 h at room temperature), and the goat anti-mouse IgG (H+L) secondary antibody (Alexa Fluor™ 488) (dilution 1:500 in 0.1% BSA, treatment for 45 min at room temperature). Nuclei were counterstained with DAPI (300 nM in 0.1% BSA, 5 min staining at room temperature). The cells were washed three times with PBS before and after the application of these steps. At the end of the experiments, the cells were photographed with the Lionheart FX automated microscope, and the taken images were analyzed in Gen5™ Microplate Data Collection and Analysis Software (Version 3.14). Apoptotic index was determined in DAPI-stained cells and calculated by applying the following formula:

$$\mathrm{Apoptotic}\;\mathrm{index}\;(\%)=\frac{number\;of\;nuclei\;with\;an\;apoptotic\;morphology}{total\;number\;of\;nuclei}\;\times \;100$$

The experiment was conducted in triplicate. Control indicates cells without GEN, DOX, or GEN–DOX treatment.

### 2.9. Caspase-3/7 and Caspase-9 Activity Measurement

For these assays, 10^4^ of SK-MEL-28 cells were cultured in each well of white, opaque 96-well plates and treated with GEN 10 µM, DOX (0.5 and 1 µM), and GEN 10 µM + DOX (0.5 and 1 µM) for 24 h. At the end of the treatment period, the plates were equilibrated at room temperature for 30 min, and 100 μL of Caspase-Glo reagents (3/7 and 9, respectively, prepared according to the manufacturer’s indications) were added to each well. The plates were shaken for 30 s and incubated for 1 h before the luminescence was read on Cytation 5. The experiment was conducted in triplicate. Control indicates cells without GEN, DOX, or GEN–DOX treatment.

### 2.10. ROS Quantification

ROS measurement was performed after the treatment of SK-MEL-28 with GEN 10 µM, DOX (0.5 and 1 µM), and GEN 10 µM + DOX (0.5 and 1 µM) using the ROS-Glo™ H_2_O_2_ kit, according to the manufacturer’s protocol. Briefly, the SK-MEL-28 cells were cultured at a density of 10^4^ cells/well in white, opaque 96-well plates and exposed to the test compounds for 18 h. Next, the H_2_O_2_ substrate solution (20 µL) was added in each well, and the plates were incubated for another 6 h. Finally, the ROS-Glo™ Detection Solution was added (100 µL/well), and the plates were kept at room temperature for 20 min. The luminescence values were read on Cytation 5. The experiment was conducted in triplicate. Control indicates cells without GEN, DOX, or GEN–DOX treatment.

### 2.11. In Ovo Irritation Assay

The potential irritant effect of the GEN 10 µM + DOX 1 µM treatment was evaluated using the CAM of fertilized chicken eggs on the 10th day of incubation. Comparatively, the strong irritant sodium lauryl sulfate (1%) was used as positive control, and distilled water was considered as negative control. The tested GEN 10 µM + DOX 1 µM sample was obtained by diluting the concentrated solutions prepared in DMSO with distilled water. The final DMSO concentration was kept below 0.5% to avoid any potential vascular toxicity from this solvent. The irritant effect was assessed by applying the aforementioned samples directly on the CAM and noting the time of the appearance of hemorrhage (H), lysis (L), or coagulation (C) during a five-minute interval. Representative images from T0 and T5 time points were taken using the Discovery 8 SteREO microscope and processed in the ZEN core version 3.8 software (Zeiss, Göttingen, Germany). The irritation score was calculated using the formula mentioned by Cozma et al. [33]. The experiments were conducted in triplicate. Distilled water was used as negative control, while SLS 1% was selected as positive control.

### 2.12. Statistical Analysis

The results were statistically analyzed using GraphPad Prism software (GraphPad Software, San Diego, CA, USA, www.graphpad.com), Version 9.3.1. The differences between the GEN, DOX, and GEN–DOX treated groups and control (cells without treatment) were determined by applying one-way ANOVA and Dunnett’s multiple comparison tests. All statistically significant results were marked using “*”, as follows: * *p* < 0.05; ** *p* < 0.01; *** *p* < 0.001; **** *p* < 0.0001.

## 3. Results

### 3.1. GEN–DOX Treatment Triggers a Selective Cytotoxicity in SK-MEL-28 Cells

#### 3.1.1. Impact of GEN–DOX Treatment on Cell Viability

**Figure 1:**
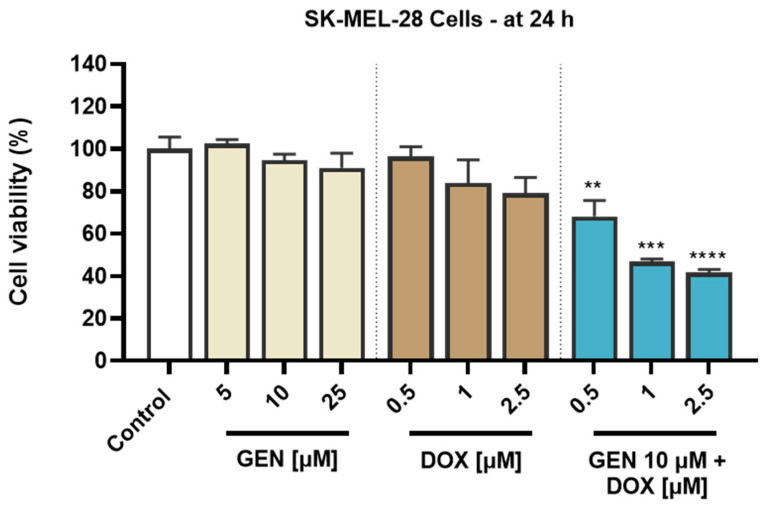
Evaluation of the influence of genistein (GEN; 5, 10, 25 µM), doxorubicin (DOX; 0.5, 1, 2.5 µM), and genistein (GEN) 10 µM - doxorubicin (DOX; 0.5, 1, 2.5 µM) treatments on the viability of SK-MEL-28 cells after 24 h. The results are presented as viability percentages (%) normalized to control (cells without GEN, DOX, or GEN-DOX treatment) and represent mean values ± standard deviation of three experiments performed in triplicate. The statistical differences between groups were verified by applying the one-way ANOVA analysis and Dunnett’s multiple comparisons post-test. Statistical significance is marked with “*” (** *p* < 0.01; *** *p* < 0.001 and **** *p* < 0.0001 versus control).

To investigate the cytotoxic potential of GEN, DOX, and their combinatorial treatment in SK-MEL-28 CM cells and healthy HaCaT cells, an MTT assay was performed following 24 h of treatment. As evidenced in Figure 1, both GEN and DOX caused a concentration-dependent reduction in the viability of SK-MEL-28 cells. The cell viability percentages determined after the SK-MEL-28 cells’ exposure to GEN were 102.47%, 94.63%, and 91.11% at the tested concentrations of 5, 10, and 25 µM. DOX 0.5 µM reduced the viability to 96.37%, while at 1 and 2.5 µM to 83.9% and 79.03%, respectively. The addition of GEN 10 µM to DOX (0.5, 1, and 2.5 µM) caused a massive and significant reduction in the SK-MEL-28 cells’ viability to values under 70%, all percentages for the GEN + DOX combinations being lower compared to the ones obtained after the cells’ treatment with GEN or DOX individually and reaching statistical significance compared to control. Specifically, the registered viabilities for GEN 10 µM + DOX 0.5 µM, GEN 10 µM + DOX 1 µM, and GEN 10 µM + DOX 2.5 µM associations were 68.05%, 46.73%, and 41.60%, respectively.

**Figure 2:**
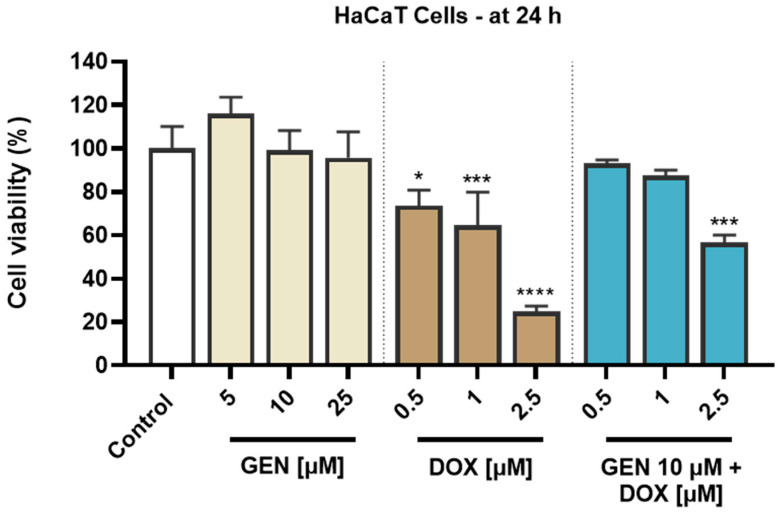
Evaluation of the influence of genistein (GEN; 5, 10, 25 µM), doxorubicin (DOX; 0.5, 1, 2.5 µM), and genistein (GEN) 10 µM - doxorubicin (DOX; 0.5, 1, 2.5 µM) treatments on the viability of HaCaT cells after 24 h. The results are presented as viability percentages (%) normalized to control (cells without GEN, DOX, or GEN–DOX treatment) and represent mean values ± standard deviation of three experiments performed in triplicate. The statistical differences between groups were verified by applying the one-way ANOVA analysis and Dunnett’s multiple comparisons post-test. Statistical significance is marked with “*” (* *p* < 0.05; *** *p* < 0.001 and **** *p* < 0.0001 versus control).

An opposite effect was obtained in HaCaT cells (Figure 2). GEN induced no cytotoxic effects at the tested concentrations of 5, 10, and 25 µM, as the viability reached values of 116.07%, 99.22%, and 95.77% after the 24 h treatment, indicating a slight stimulatory effect at the lowest concentration and a dose-dependent reduction in viability at concentrations equal to and higher than 10 µM. Comparatively, DOX was found to induce a significant cytotoxicity in HaCaT cells after 24 h of treatment, as even at the lowest concentration tested, the viability was <80%. The most significant reductions were obtained at 2.5 µM (24.96%). A significant decrease in cell viability compared to control (to 56.70%) was also obtained when GEN 10 µM was associated with DOX 2.5 µM. However, when GEN 10 µM was added to DOX 0.5 and 1 µM, the viability of HaCaT cells was 92.98% and 87.57%. Overall, the viability values for the GEN–DOX treatment were higher compared to the ones for the individual DOX treatment, suggesting the ability of GEN to reverse the cytotoxicity of DOX in this cell line.

The solvents used for the preparation of GEN and DOX stock solutions had no impact on the viability of SK-MEL-28 and HaCaT cells. 

#### 3.1.2. Combination Index Calculation for GEN–DOX Treatment

To assess whether the interaction between GEN and DOX in SK-MEL-28 and HaCaT cells is synergistic, additive, or antagonistic, the combination index (CI) values were calculated using the Chou–Talalay method based on the viability results presented in Section 3.1.1. In the case of SK-MEL-28 cells, as presented in Table 1, the association of GEN (10 µM) with all concentrations of DOX resulted in synergistic interactions that were strong only when GEN 10 µM was combined with DOX 0.5 and 1 µM. Conversely, in HaCaT cells, GEN antagonized the cytotoxicity of DOX in all applied treatment regimens. Strong antagonism was observed for the GEN 10 µM + DOX 0.5 µM and GEN 10 µM + DOX 1 µM treatments, and antagonism was obtained for GEN 10 µM + DOX 2.5 µM association.

#### 3.1.3. Impact of GEN–DOX Treatment on Cell Morphology and Confluence

**Figure 3:**
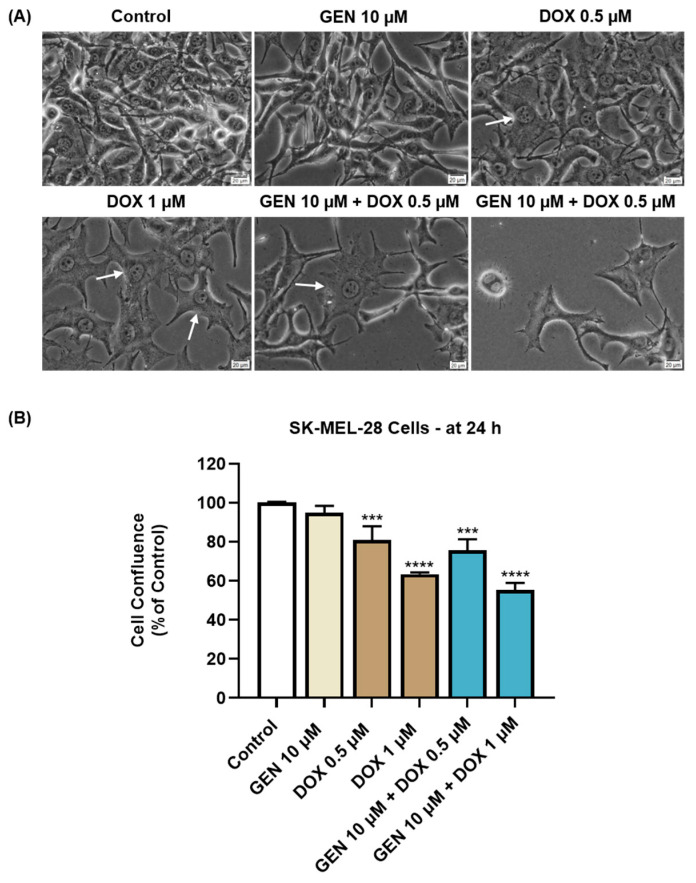
(**A**) Morphology of SK-MEL-28 cells exposed to GEN (10 µM), DOX (0.5 and 1 µM), and GEN (10 µM) + DOX (0.5 and 1 µM) for 24 h. The scale bars indicate 20 µm. The white arrows indicate cell hypertrophy. (**B**) Cell confluence determination in SK-MEL-28 cells exposed to GEN (10 µM), DOX (0.5 and 1 µM), and GEN (10 µM)- DOX (0.5 and 1 µM) for 24 h. The results are presented as percentages (%) normalized to control (cells without GEN, DOX, or GEN–DOX treatment) and represent mean values ± standard deviation of three experiments performed in triplicate. The statistical differences between groups were verified by applying the one-way ANOVA analysis and Dunnett’s multiple comparisons post-test. Statistical significance is marked with “*” (*** *p* < 0.001; **** *p* < 0.0001 versus control).

The impact of GEN 10 µM, DOX (0.5 and 1 µM), and GEN 10 µM + DOX (0.5 and 1 µM) on the morphology and confluence of SK-MEL-28 and HaCaT cells was next investigated. With regard to the morphological aspect of SK-MEL-28 cells (Figure 3A), GEN 10 µM induced elongation compared to control, while DOX 0.5 and 1 µM caused cell hypertrophy (white arrows) evidenced by an increase in cell size. Comparatively, the combination treatment with GEN 10 µM and DOX 0.5 µM caused hypertrophy and cell elongation, while the one with GEN 10 µM and DOX 1 µM induced cell shrinkage and rounding. The confluence of SK-MEL-28 cells (Figure 3B) was reduced by all applied treatments; however, statistical significance was reached only for DOX (0.5 µM and 1 µM) and GEN (10 µM) + DOX (0.5 µM and 1 µM). The lowest confluence (55.38%) was obtained when GEN 10 µM was combined with DOX 1 µM.

**Figure 4:**
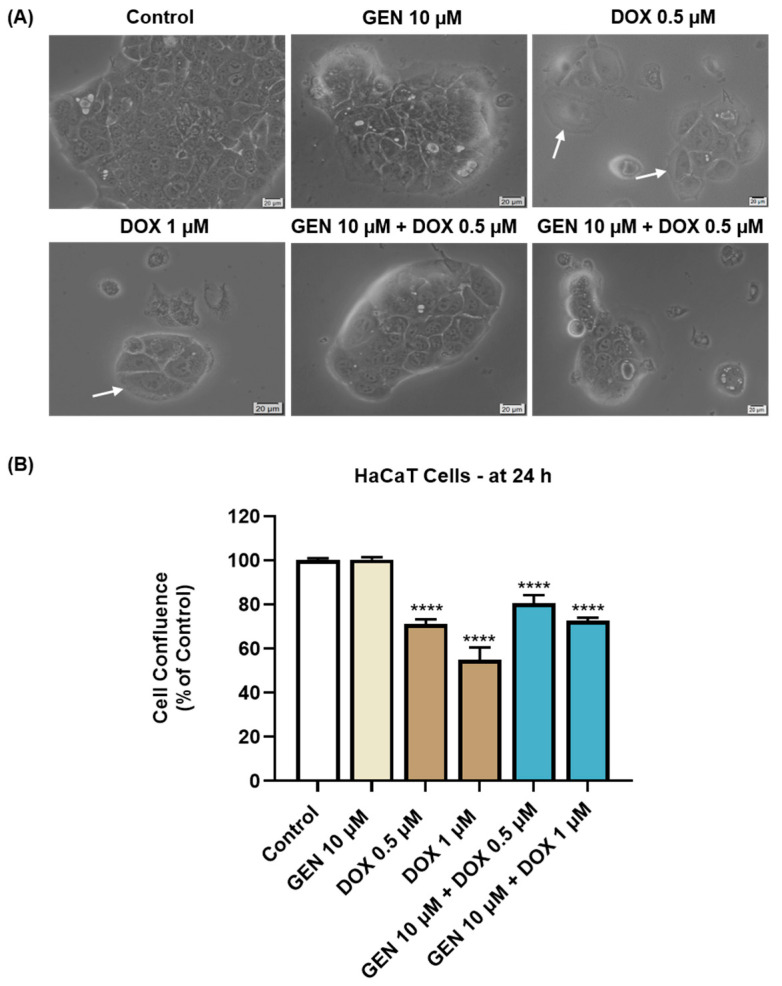
(**A**) Morphology of HaCaT cells exposed to GEN (10 µM), DOX (0.5 and 1 µM), and GEN (10 µM) + DOX (0.5 and 1 µM) for 24 h. The scale bars indicate 20 µm. The white arrows indicate cell hypertrophy. (**B**) Cell confluence determination in HaCaT cells exposed to GEN (10 µM), DOX (0.5 and 1 µM), and GEN (10 µM)-DOX (0.5 and 1 µM) for 24 h. The results are presented as percentages (%) normalized to control (cells without GEN, DOX, or GEN–DOX treatment) and represent mean values ± standard deviation of three experiments performed in triplicate. The statistical differences between groups were verified by applying the one-way ANOVA analysis and Dunnett’s multiple comparisons post-test. Statistical significance is marked with “*” (**** *p* < 0.0001 versus control).

As presented in Figure 4A, GEN 10 µM caused no morphological changes in HaCaT cells compared to control, while hypertrophy and rounding were noticed after the HaCaT cells’ treatment with DOX 0.5 µM and 1 µM. This cytotoxic signs were considerably alleviated after the addition of GEN 10 µM to DOX treatments. The confluence of HaCaT cells (Figure 4B) was also impaired by their treatment with DOX (0.5 µM and 1 µM), reaching values lower than 75%. GEN 10 µM suppressed the impact of DOX on HaCaT cells’ confluence, with the percentages being higher for the GEN + DOX associations compared to the DOX-only exposures.

The solvents used for the preparation of GEN and DOX stock solutions caused no impact of the cells’ morphology or confluence.

#### 3.1.4. Impact of GEN–DOX Treatment on Nuclear and Cytoskeletal Reorganization

**Figure 5:**
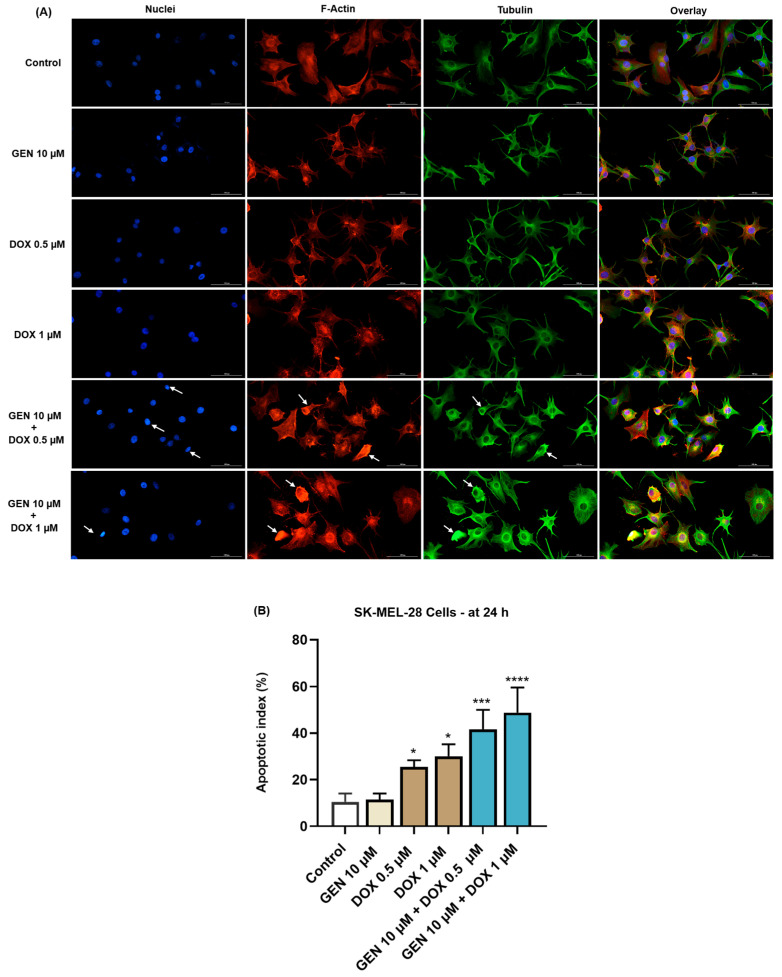
(**A**) Immunofluorescence visualization of nuclei, F-actin, and tubulin in SK-MEL-28 cells exposed for 24 h to GEN 10 µM, DOX 0.5 µM, DOX 1 µM, and GEN 10 µM - DOX (0.5 and 1 µM). The scale bars indicate 100 µm. The white arrows indicate apoptotic-like nuclear and cytoskeletal changes. (**B**) Calculated apoptotic index values in SK-MEL-28 cells exposed for 24 h to GEN 10 µM, DOX 0.5 µM, DOX 1 µM, and GEN 10 µM-DOX (0.5 and 1 µM). The results are presented as percentages (%) and represent mean values ± standard deviation of three experiments performed in triplicate. The statistical differences between groups were verified by applying the one-way ANOVA analysis and Dunnett’s multiple comparisons post-test. Statistical significance is marked with “*” (* *p* < 0.05; *** *p* < 0.001; **** *p* < 0.0001 versus control).

Next, the effect of SK-MEL-28 and HaCaT cells’ treatment with GEN 10 µM, DOX 0.5 µM, DOX 1 µM, and their combinations on the morphology of cell nuclei and distribution of F-actin and tubulin filaments was evaluated. As indicated by the results from Figure 5A, no significant changes in the aspect of these cellular components or condensations were caused by the individual treatment with GEN 10 µM, although the cells appeared more elongated in shape compared to control. DOX (0.5 and 1 µM) induced nuclear constriction but no evident alterations in the distribution of F-actin and tubulin filaments. Some cells treated with DOX at these concentration presented an increased size, indicating hypertrophy. Considerable condensations of nuclear chromatin, F-actin, and tubulin were observed after the cells’ exposure to GEN 10 µM + DOX 0.5 µM and GEN 10 µM + DOX 1 µM. These changes were accompanied by cell rounding. An elevated apoptotic index was obtained for SK-MEL-28 cells (Figure 5B) exposed for 24 h to the individual treatments with DOX 0.5 µM and 1 µM; however, the highest values were registered after the addition of GEN 10 µM.

**Figure 6:**
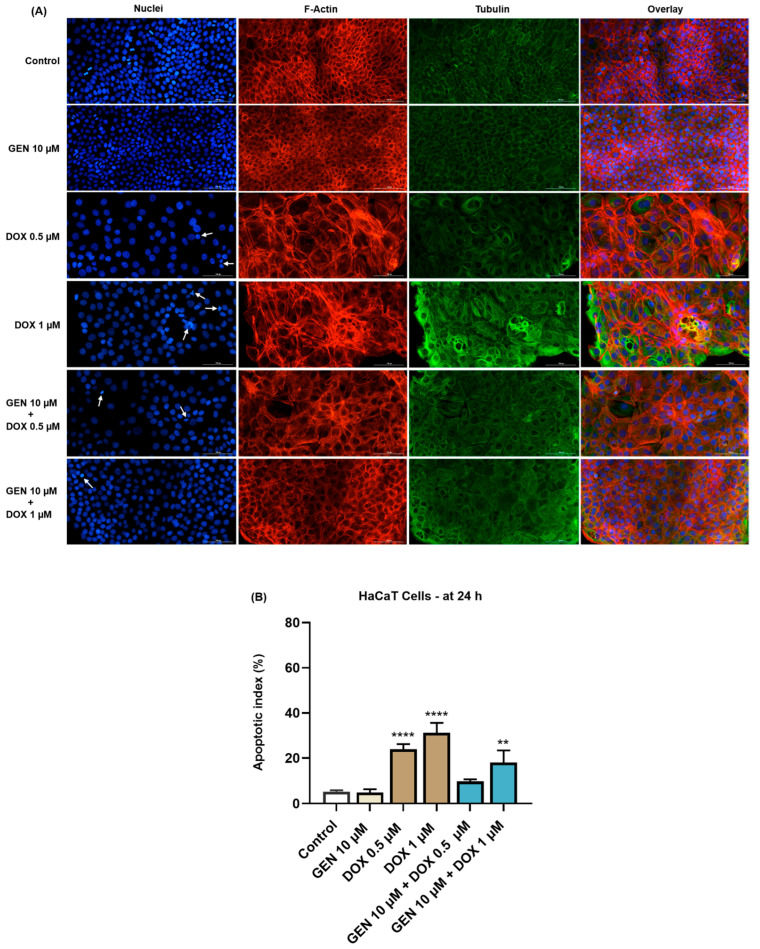
(**A**) Immunofluorescence visualization of nuclei, F-actin, and tubulin in HaCaT cells exposed for 24 h to GEN 10 µM, DOX 0.5 µM, DOX 1 µM, and GEN 10 µM + DOX (0.5 and 1 µM). The scale bars indicate 100 µm. The white arrows indicate apoptotic-like nuclear changes. (**B**) Calculated apoptotic index values in HaCaT cells exposed for 24 h to GEN 10 µM, DOX 0.5 µM, DOX 1 µM, and GEN 10 µM + DOX (0.5 and 1 µM). The results are presented as percentages (%) and represent mean values ± standard deviation of three experiments performed in triplicate. The statistical differences between groups were verified by applying the one-way ANOVA analysis and Dunnett’s multiple comparisons post-test. Statistical significance is marked with “*” (** *p* < 0.01; **** *p* < 0.0001 versus control).

In the case of HaCaT cells (Figure 6A), GEN 10 µM did not affect the aspect of nuclei or cytoskeletal fibers compared to control. Massive condensation of chromatin and F-actin was noticed after their treatment with DOX 0.5 µM. Comparatively, DOX at the concentration of 1 µM caused condensation, fragmentation, and deformation of the cells’ nuclei accompanied by the constriction of both F-actin and tubulin fibers. These alterations were also accompanied by an elevated cell size caused by DOX. The addition of GEN 10 µM to DOX treatments alleviated the changes induced by this chemotherapy drug at nuclear and cytoskeletal levels, and also reduced the apoptotic index values, which were significantly increased in the DOX-treated cells (Figure 6B).

### 3.2. GEN–DOX Treatment Activates Intrinsic Apoptosis and Generates Oxidative Stress in SK-MEL-28 Cells

#### 3.2.1. Impact of GEN–DOX Treatment on Caspase-3/7 and -9 Activation

**Figure 7:**
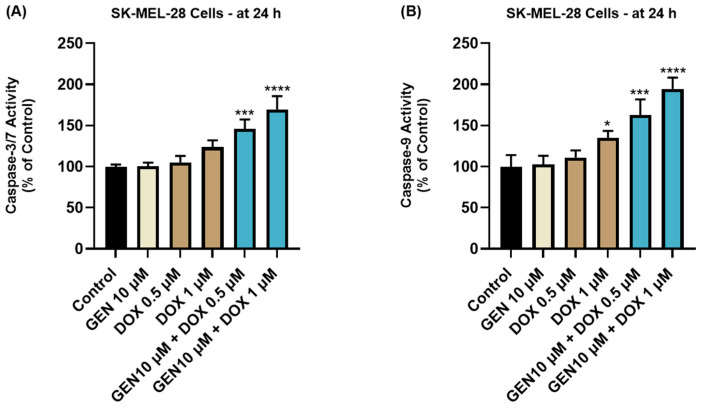
Evaluation of the influence of GEN 10 µM, DOX 0.5 µM, DOX 1 µM, and GEN 10 µM - DOX (0.5 and 1 µM) on the activity of (**A**) Caspase-3/7 and (**B**) Caspase-9 in SK-MEL-28 cells after 24 h of treatment. The results are presented as percentages (%) normalized to control (cells without treatment) and represent mean values ± standard deviation of three experiments performed in triplicate. The statistical differences between groups were verified by applying the one-way ANOVA analysis and Dunnett’s multiple comparisons post-test. Statistical significance is marked with “*” (* *p* < 0.05; *** *p* < 0.001; **** *p* < 0.0001).

The impact of GEN 10 µM, DOX 0.5 µM, DOX 1 µM, and GEN 10 µM + DOX (0.5 and 1 µM) treatments on the activity of Caspases-3/7 and -9 in SK-MEL-28 cells is presented in Figure 7. The 24 h treatment with GEN 10 µM induced no modifications in the activity of Caspase-3/7 or -9 compared to control. On the contrary, DOX (0.5 µM and 1 µM) treatments increased Caspase-3/7 activity to around 105% and 120%, and elevated Caspase-9 activity to 111% and 134%, respectively. The most significant increases in the activities of both caspases (at percentages higher than 140%) were obtained after the SK-MEL-28 cells’ exposure to GEN 10 µM+DOX 0.5 µM and GEN 10 µM+DOX 1 µM.

#### 3.2.2. Impact of GEN–DOX Treatment on Intracellular ROS Production

**Figure 8:**
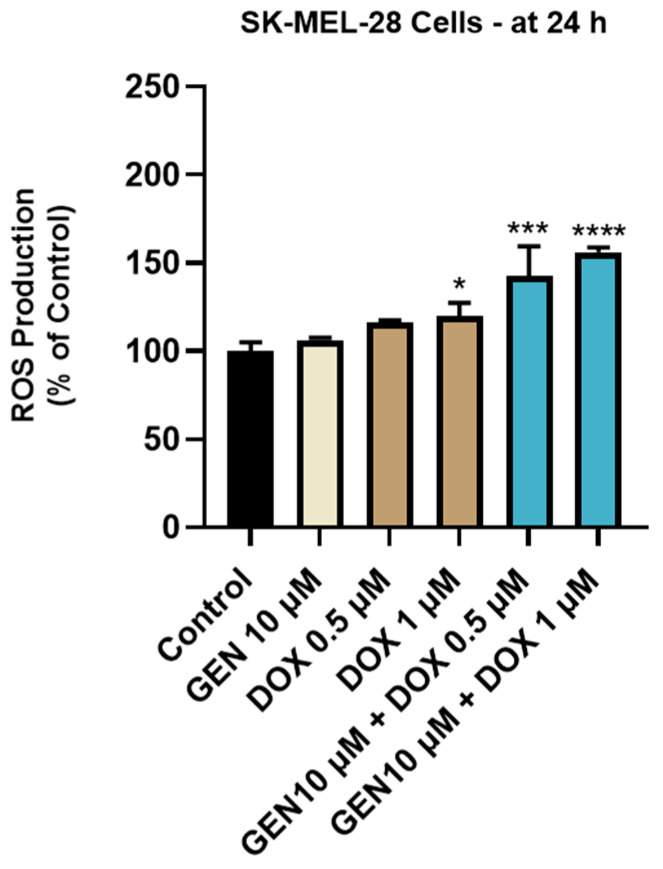
Evaluation of the influence of genistein GEN 10 µM, DOX 0.5 µM, DOX 1 µM, and GEN 10 µM - DOX (0.5 and 1 µM) on reactive oxygen species (ROS) production in SK-MEL-28 cells after 24 h of treatment. The results are presented as percentages (%) normalized to control (untreated cells) and represent mean values ± standard deviation of three experiments performed in triplicate. The statistical differences between groups were verified by applying the one-way ANOVA analysis and Dunnett’s multiple comparisons post-test. Statistical significance is marked with “*” (* *p* < 0.05; *** *p* < 0.001; **** *p* < 0.0001 versus control).

The impact of GEN 10 µM, DOX 0.5 µM, DOX 1 µM, and GEN 10 µM + DOX (0.5 and 1 µM) treatments on intracellular oxidative stress was finally investigated. As observed in Figure 8, a significant increase in ROS production was obtained after the SK-MEL-28 cells treatment with DOX 1 µM. However, the highest increase in the intracellular ROS level (to 143% and 156%) was caused by the 24 h treatment with GEN (10 µM) + DOX (0.5 and 1 µM).

### 3.3. GEN–DOX Treatment Lacks Irritant Effect in Ovo

**Figure 9:**
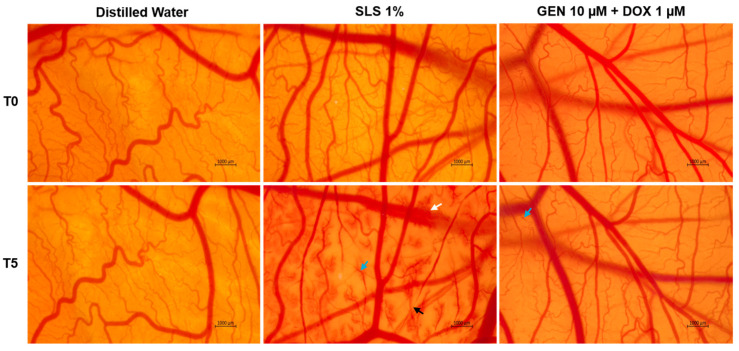
The impact of GEN 10 µM - DOX 1 µM treatment on the vascular structure of the chorioallantoic membrane (CAM) of fertilized chicken eggs. The images at T0 were taken before the application of the treatments, while the ones at T5 were taken 5 min after application. The scale bars indicate 1000 µm. The arrows indicate vascular damage (white—hemorrhage, blue—coagulation, and black—lysis).

As part of its safety profile, the potential irritant effect of GEN 10 µM + DOX 1 µM treatment was finally assessed. As observed in Figure 9, massive vascular damage was obtained only after the contact of the CAM with the positive control, SLS 1%, that caused bleeding, vessel lysis, and coagulation. Distilled water induced no toxicity on the CAM. With regard to the GEN 10 µM + DOX 1 µM association, it caused only slight coagulation within some blood vessels after treatment, without any signs of hemorrhage or lysis.

The irritation scores are presented in Table 2. According to the obtained results, the GEN 10 µM + DOX 1 µM sample was classified as non-irritant on the CAM, similar to distilled water, while SLS 1% was categorized as a strong irritant.

## 4. Discussion

CM represents a melanocytic cancer that, despite its rarity, stands as the most aggressive skin neoplasia, with limited treatment outcomes at present due to its intrinsic or acquired resistance which still remains a problem to be resolved [34]. DOX stands as a major component of the current chemotherapy regimens and one of the most effective anti-tumor medications ever approved for clinical use [12]. However, its utilization in CM treatment remains limited by drug resistance and severe adverse reactions [5,12]. Considering these aspects comprehensively, many successful initiatives have attempted to improve the anti-tumor activity of DOX against CM by resorting to combinatorial treatments that reverse resistance and alleviate toxicity [14,15,16,17]. A particular interest in this regard has been given to natural products that, when associated with DOX, produce an enhanced anti-melanoma effect and reduced toxic activities [6,22]. GEN is the most abundant soy-derived isoflavone, with proven anti-neoplastic activities and the potential in enhancing the efficacy while ameliorating the toxicity of chemotherapy agents [35,36]. Preceding studies extensively presented the synergistic interaction between GEN and DOX in different cancers (e.g., breast, prostate), leading to improved treatments [28,29], while their combinatorial treatment has not been investigated in CM yet, to the best of our knowledge. Therefore, in the present study, we explored the combinatorial treatment between the conventional chemotherapeutic DOX and the phytocompound GEN in terms of anti-neoplastic activity against CM cells and safety in vitro and in ovo profile.

The tested concentrations of GEN (5, 10, and 25 µM) and DOX (0.5, 1, and 2.5 µM) were selected based on a comprehensive review of some representative studies in which their anti-tumor activity was assessed [37,38,39,40]. The anti-CM activity of GEN, DOX, and GEN–DOX treatments was explored in the SK-MEL-28 cell line that was chosen as a representative experimental model for CM, as it is suitable for applications in cancer or toxicology research, presents the BRAF^V600E^ driver mutation, achieves similar histopathological characteristics to CM in vivo, and is described as a CM cell line that presents resistance to DOX treatment [41,42,43]. This study also proposed not only the evaluation of the potential toxicity of the GEN–DOX combination in a healthy-skin-derived cell line, but also the assessment of the potential cytoprotective activity of GEN against DOX-induced cellular damage. This part of the study employed the HaCaT cells as 2D in vitro models, which resemble isolated keratinocytes with regard to their surface markers, morphological features, and functionalities, reconstruct a well-defined epidermis after in vivo inoculation, and are widely used for toxicological studies [30,44,45]. This work was structured into three distinct parts, as follows: (i) the evaluation of the potential selective cytotoxicity of GEN–DOX treatment in SK-MEL-28 cells compared to HaCaT cells; (ii) the mechanistic insight into the anti-tumor effects of GEN–DOX in SK-MEL-28 cells with regard to intrinsic apoptosis and ROS generation; and (iii) the assessment of the potential irritant effect of GEN–DOX on the chorioallantoic membrane. The findings collected throughout this study outlined three main novelties, as follows: (i) the addition of GEN to DOX led to an improved cytotoxic effect in SK-MEL-28 CM cells compared to the individual treatments, due to the synergistic interaction between the two anti-tumor agents; (ii) the anti-neoplastic property exerted by the GEN–DOX treatment in SK-MEL-28 CM cells was related to the ability of this therapeutic association to reduce cell viability, damage cell morphology, cause apoptotic-specific nuclear impairments, promote tubulin and F-actin constriction, augment caspases-3/7 and -9 activities, and increase intracellular ROS levels; (iii) GEN antagonized the cytotoxic effect of DOX in HaCaT keratinocytes, reducing its cytotoxic properties in this healthy cell line; and (iv) the GEN–DOX treatment lacked irritant effect in ovo.

This study debuted with a comparative evaluation of the cytotoxic effects retained by GEN, DOX, and their combinatorial treatment in SK-MEL-28 and HaCaT cells. The main findings (Figure 1) illustrated that GEN and DOX caused a concentration-dependent decrease in the viability of SK-MEL-28 cells, but without reaching statistical significance. These observations are supported by preceding reports. For instance, Cui et al. found that GEN induced a time- and dose-dependent inhibition in B16F10 CM cells’ viability that reached statistical significance only at 100 µM after 24 and 48 h of treatment, while lower concentrations (12.5–50 μM) caused no considerable cytotoxicity [25]. DOX showed significant toxicity in B16F10 and B16-OVA CM cells after 72 h of treatment with IC_50_ values of 0.24 and 0.35 μM, respectively [46]. Intriguingly, GEN elevated the cytotoxic effect of DOX in SK-MEL-28 cells, with the viability values obtained after the cells’ exposure to GEN–DOX being lower compared to the ones registered following the individual treatments. To the best of our knowledge, the augmented anti-tumor effect of GEN–DOX against CM cells has not been discussed yet. However, the ability of GEN to increase the chemotherapeutic effect of DOX was previously revealed: Xue and colleagues showed that GEN (30 µM) enhanced the cytotoxicity of DOX (0.07, 0.7, 7, or 70 µM) in MCF-7/Adr drug-resistance breast cancer cells [28], while Ikawati et al. presented the efficacy of GEN (at a low and a high concentration—1 µM and 50 µM, respectively) in augmenting the toxic effects of DOX (10 nM) in 4T1 triple-negative breast cancer metastatic cells after 24, 48, and 72 h of treatment [47]. With regard to HaCaT cells, GEN (5, 10, and 25 µM) was not cytotoxic, while DOX significantly affected their viability at all tested concentrations (Figure 2). The toxic properties of DOX in healthy cells (i.e., HOK oral keratinocytes, HGF gingival fibroblast, HPLF periodontal ligament fibroblast, and HPC pulp cell), at concentrations covering the ones tested in the present study and higher were also previously documented [48]. The high sensitivity of HaCaT cells to DOX and other topoisomerase inhibitors has been presented as well [49]. More recently, Yokomichi et al. reported a reduction in the survival rate of HaCaT keratinocytes and Normal Human Dermal Fibroblasts after a 24 h exposure of these cells to DOX concentrations ranging between 0.1 and 10 µM [50]. Luanpitpong et al. elaborated on the underlying mechanisms of DOX-induced toxicity in HaCaT cells, demonstrating the ability of this chemotherapy drug to trigger mitochondrial apoptotic pathway and induce oxidative stress after 24 h of treatment with concentrations similar to the ones tested in this study [51]. Interestingly, GEN (10 µM) reversed the cytotoxic properties of DOX (0.5 µM and 1 µM) in HaCaT cells at all combination treatments, with the viabilities reaching higher percentages compared to the DOX-only exposures. The cytoprotective ability of GEN against DOX-caused toxicity was also mentioned in the literature. Bai Z and Wang Z showed that GEN confers protection against DOX-induced cardiotoxicity in vivo by downregulating apoptosis activation, suppressing serum cardiac troponin, and reducing redox and pro-inflammatory markers [52]. Based on these findings, the interaction type between GEN and DOX in SK-MEL-28 and HaCaT cells was further determined using the Chou–Talalay method (Table 1), the most commonly employed approach for the quantification of drug combinations. It allows the differentiation between synergistic, additive, or antagonistic interactions of combined compounds based on the determined CI [53,54], and was previously employed for the evaluation of interaction types in combination therapies containing either GEN or DOX [55,56]. Intriguingly, strong synergism was obtained in SK-MEL-28 cells and strong antagonism was observed in HaCaT cells only for the treatments of GEN 10 µM + DOX 0.5 µM and GEN 10 µM + DOX 1 µM, while the last combinatorial therapy encompassing GEN 10 µM and DOX 2.5 µM led to synergism and antagonism, respectively. Considering these findings, the combinatorial treatment investigated further throughout the study—GEN 10 μM + DOX (0.5 µM and 1 µM)—comprised the associations that exerted a strong interaction in SK-MEL-28 and HaCaT cells and the concentrations that can be physiologically achieved following GEN and DOX clinical administration [57,58].

As part of the cytotoxicity profile of GEN, DOX, and GEN-DOX treatments, the morphology and confluence of SK-MEL-28 and HaCaT cells were evaluated as well (Figure 3 and Figure 4). The interesting on-topic discovery was that GEN 10 µM caused cell elongation only in SK-MEL-28 cells, with no morphology impairment in HaCaT cells, while DOX triggered visible hypertrophy in both CM cells and keratinocytes. In agreement with this observation stand other studies reporting that GEN produces alterations in B16F10 CM cells which became elongated in shape and presented slim pseudopodia-like protrusions [25], and that DOX induces cell hypertrophy in H9c2 rat cardiac cells [59]. GEN 10 µM was found to enhance the morphological and confluence alterations caused by DOX 0.5 and 1 µM, respectively, in SK-MEL-28 cells, and to alleviate these effects in HaCaT cells. Additionally, the treatment of SK-MEL-28 cells with GEN 10 µM associated with DOX 0.5 and 1 µM (Figure 5) caused significant changes in nuclear morphology and distribution of cytoskeletal filaments characterized by chromatin constriction and condensation of F-actin and tubulin fibers leading to cell deformation and rounding, which were considerably higher compared to control or GEN- and DOX-only treatments. These signs are consistent with apoptosis, a programmed cell death accompanied by several stereotypic series of events, causing specific intracellular restructuring, which affects both the nuclear and the cytoplasmic compartments [60]. Chromatin usually transitions from a heterogeneous network to a constricted form that is further fragmented into apoptotic bodies [61], while cytoskeletal tubulin and actin reorganize into thick, condensed bundles [60,62]. Conversely, in HaCaT cells (Figure 6), the massive contraction of nuclei and cytoskeletal fibers induced by DOX 0.5 and 1 µM were abolished by the addition of GEN 10 µM.

To further confirm the pro-apoptotic properties of GEN–DOX treatments in SK-MEL-28 cells, their efficacy in modulating the activity of caspases-3/7 and -9 was explored (Figure 7). Caspases, which are broadly divided into initiators (e.g., Caspase 9) and effectors (e.g., Caspases 3, 7), play a primordial role in the events occurring during apoptosis [63]. Caspase 3 is directly involved in the apoptotic-specific morphological alterations and DNA fragmentation, while caspase 7 has a higher relevance for cell viability loss [64]. Caspase 9 significantly contributes to the intrinsic (mitochondrial) apoptotic pathway which is triggered by numerous stimuli such as chemotherapy agents, radiation, or stress [65]. Once activated, it can subsequently activate caspases 3 and 7 [66]. The combination therapy of SK-MEL-28 cells with GEN (10 µM) and DOX (0.5 µM and 1 µM) led to a significant increase in the activity of both caspase types, which was higher compared to the GEN- or DOX-individual treatments. The association between GEN and DOX was also reported to induce apoptosis and cell cycle arrest in breast cancer cells [28]. Finally, the influence of GEN–DOX therapy on intracellular ROS levels in SK-MEL-28 cells was assessed. ROS represent a group of highly reactive molecules that, in moderate quantities, contribute to several cellular functions, but when increased, participate in pathological conditions such as tumor initiation and progression [67]. Enhanced levels of ROS also damage intracellular components and organelles and trigger programmed cell death types such as apoptosis [68]. As shown in Figure 8, a significant increase in ROS production in SK-MEL-28 cells after 24 h of treatment was obtained for the GEN (10 µM) + DOX (0.5 µM and 1 µM) associations, which were higher compared to control or the individual treatments. Amplified oxidative stress was also detected in RM-1 prostate cancer cells following their concomitant exposure to GEN- and DOX-loaded polypeptide nanoparticles [29].

Lastly, this study included the safety assessment of the GEN 10 µM + DOX 1 µM association in ovo, in terms of vascular toxicity and irritant potential, using the CAM of chicken embryo as an experimental model. CAM presents a highly vascularized structure, formed of veins, arteries, and capillaries, which allows the assessment of the toxic and irritant potential of substances applied locally [69,70]. The results (Figure 9, Table 2) suggested a lack of toxic effects with regard to this combinatorial treatment, apart from very slight signs of coagulation. Based on the irritation score, the GEN 10 µM + DOX 1 µM association was classified as a non-irritant on the CAM. As far as we are aware, this combinatorial treatment has not been previously studied in ovo in terms of irritant effect. However, at a slightly higher concentration (1.7 µM), DOX was described as non-irritant on the CAM [71]. Other work reported that the exposure of the CAM to GEN (50 µM) and its association with aspirin produced no signs of vascular irritation, such as hemorrhage, lysis, or coagulation [72].

Collectively, these novel findings stress the safety and improved anti-melanoma efficacy of the GEN–DOX combinatorial treatment at clinically obtainable concentrations and in representative in vitro and in ovo models. While this study reports important results on the potential combination therapy comprising GEN and DOX for CM therapy, certain limitations should be addressed in future investigations. Firstly, although SK-MEL-28 cells are a proper CM model for this study due to their features and resistance to DOX, expanding these analyses to additional CM cell lines would strengthen the generalizability of the findings presented herein. Secondly, this study presented the interactions between GEN (10 µM) and DOX (0.5 and 1 µM) at physiological concentrations, while the investigations at the half-maximal inhibitory concentration (IC_50_) of GEN and DOX in these cell lines would bring additional value to their utilization in CM treatment. Thirdly, the interaction type between GEN and DOX was assessed using the most commonly employed technique for drug interactions, the Chou–Talalay method, while complementary advanced approaches (e.g., isobolographic analysis) should further validate the nature of their interaction. Lastly, the clinical translation of the efficacy of this association in CM treatment should be further confirmed in vivo.

## 5. Conclusions

The findings discovered throughout this study concluded that GEN increased the sensitivity of SK-MEL-28 CM cells to DOX, with their association leading to an improved anti-neoplastic activity compared to the individual treatments, while also abolishing cytotoxicity and hypertrophy in HaCaT keratinocytes. Mechanistically, GEN enhanced the apoptotic and ROS-generating ability of DOX in SK-MEL-28 cells. Their combination caused no irritant effect on the CAM.

## Figures and Tables

**Table 1:** The calculated combination index (CI) and the interaction type between genistein (GEN) and doxorubicin (DOX) in SK-MEL-28 and HaCaT cells after 24 of treatment.

Cell Line	GEN 10 µM + DOX (µM)	Combination Index (CI)	Type of Interaction
SK-MEL-28	0.5	0.192	Strong synergism
	1	0.170	Strong synergism
	2.5	0.353	Synergism
HaCaT	0.5	3.635	Strong antagonism
	1	4.191	Strong antagonism
	2.5	2.497	Antagonism

**Table 2:** Irritation score values determined for the treatments applied in this study: distilled water (negative control), sodium lauryl sulfate (SLS) 1% (positive control), and GEN 10 µM + DOX 1 µM.

Sample	Irritation Score
Distilled water	0.07
SLS 1%	19.35
GEN 10 µM + DOX 1 µM	0.28

## Data Availability

The data presented in this study are available on request from the corresponding author.

## Abbreviations

The following abbreviations are used in this manuscript:

CM	Cutaneous melanoma
DOX	Doxorubicin
GEN	Genistein
CAM	Chorioallantoic membrane
UV	Ultraviolet radiation
CTLA-4	Cytotoxic T-lymphocyte associated protein-4
PD-1	Programmed cell death protein-1
BRAF	v-Raf murine sarcoma viral oncogene homolog B1
MEK	Mitogen-activated protein kinase
GEN–DOX	Genisten–doxorubicin
DAPI	4′,6-diamidino-2-phenylindole
BSA	Bovine serum albumin
DMEM	Dulbecco’s Modified Eagle Medium
EMEM	Eagle’s Minimum Essential Medium
FBS	Fetal bovine serum
DMSO	Dimethyl sulfoxide
CI	Combination index
IC50	Half-maximal inhibitory concentration
